# Dual-band stub-loaded monopole antenna with bandwidth enhancement using weighted figure-of-merit optimization

**DOI:** 10.3389/frai.2026.1796177

**Published:** 2026-06-01

**Authors:** Jun-Jiat Tiang

**Affiliations:** Centre for Wireless Technology, CoE for Intelligent Network, Faculty of Artificial Intelligence and Engineering, Multimedia University, Persiaran Multimedia, Cyberjaya, Selangor, Malaysia

**Keywords:** antenna optimization, dual-band antenna, figure-of-merit (FoM), machine learning (ML), monopole antenna, stub-loaded antenna

## Abstract

This paper presents a compact dual-band stub-loaded T-monopole antenna optimized for WLAN (2.4 GHz) and 5–6 GHz (5G/IoT) applications using a weighted figure-of-merit (FOM) and artificial neural network (ANN) surrogate modeling. Low- and high-band stubs enable independent resonance control, achieving −10 dB impedance bandwidths of 1.7–2.7 GHz (45.5%) in the lower band and 5.1–5.9 GHz (14.5%) in the upper band, with return loss depths exceeding −20 dB at resonances. This outperforms a conventional reference design (22.2% lower/9.0% upper) and prior ML-optimized stub-loaded monopoles. The weighted FOM prioritizes upper-band performance for high-data-rate needs (weights *w_₂_* = *w_₄_* = 1.5). An ANN surrogate, trained on 210 HFSS-simulated samples, yields *R*^2^ > 0.99 (training)/>0.99 (validation), enabling rapid predictions (seconds vs. minutes per EM simulation). Radiation characteristics remain suitable (gain ~2–3.2 dBi lower/~3.5–4.3 dBi upper; efficiency >80–85%). The hybrid approach offers scalable, efficient methodology for next-generation dual-band antennas, with novelty in tunable band-prioritized FOM + ANN for legacy monopole enhancement.

## 1 Introduction

The rapid expansion of the Internet of Things (IoT) has led to a growing need for specialized antennas tailored to various electronic devices. This demand highlights the necessity for more intelligent and efficient antenna design approaches. Traditional methods, which depend on manual expertise and lengthy electromagnetic (EM) simulations, are often slow and resource intensive, particularly when optimizing complex design parameters. To overcome these challenges, machine learning (ML) presents a promising solution. ML has emerged as a powerful tool for data driven analysis and optimization, offering significant potential for enhancing antenna design processes (Chen et al., 2024; Sarker et al., 2023; Zhong et al., 2022; Wu et al., 2025; Yahya et al., 2024; Zhang et al., 2023).

Recent studies have explored antenna optimization through heuristic approaches like genetic algorithms and particle swarm optimization (Yang et al., 2021; Bora et al., 2020; Bagherkhani et al., 2025; Yu et al., 2025). These methods work by iteratively assessing candidate solutions and refining search directions to systematically enhance performance, ultimately converging toward an optimal global solution.

Machine learning (ML), on the other hand, employs diverse algorithms and analytical techniques to extract underlying mathematical patterns from data. By establishing relationships between input and output parameters, ML enables predictive modeling and data-driven decision making. Unlike traditional optimization methods that focus solely on finding global optima, ML models offer broader utility—once trained, they can generate predictions for any input within their learned domain. This adaptability proves especially advantageous when working with a single dataset to address multiple different objectives (Montaser, 2023; Singh et al., 2025; Wu et al., 2024). The hybrid weighted FOM + ANN approach innovates by enabling rapid, application-specific tradeoffs (e.g., upper-band prioritization for 5G) and >2x lower band bandwidth over heuristics like GA (Yang et al., 2021; Bora et al., 2020; Bagherkhani et al., 2025; Yu et al., 2025), which require thousands of iterations. Post-training, predictions take seconds vs. hours, with independent band control via stubs—benefits for compact IoT/5G designs.

The primary objective of this work is to expand these proposed methods to tackle more intricate antenna designs and develop scalable, efficient algorithms to address computational challenges associated with managing a large number of design parameters (Patel et al., 2025; Babu and Sree, 2024; Haque et al., 2023).

Stub loading is a widely used technique in antenna engineering to enhance impedance bandwidth and enable multiband operation without significantly increasing antenna size. A stub, typically implemented as an additional branch or parasitic strip, introduces controlled resonances that interact with the main radiator (Tirado-Méndez et al., 2025; Wu et al., 2025; Chen et al., 2024). By carefully selecting the stub length and placement, it is possible to excite additional resonant modes or broaden existing ones, thereby achieving wideband or dual band performance.

In monopole and planar antenna structures, stubs have been effectively used to cover the 2.4/5 GHz WLAN bands, as demonstrated in (Kuo and Wong, 2003), where a printed double-T monopole achieved dual band operation. Later, optimization-oriented studies such as (Sharma et al., 2020) explored tuning strategies for stub loaded monopoles using data-driven regression models. These works confirm that stubs not only enable dual band operation but also provide a compact and practical approach for matching the stringent bandwidth requirements of modern wireless systems.

Although stub loading effectively introduces the required resonances, traditional design relies heavily on manual tuning and extensive parametric simulations. To address this limitation, performance-driven optimization metrics such as the Figure of Merit (FOM) have been adopted (Ghasemi, 2025). The FOM allows antenna designs to be quantitatively assessed by combining critical factors such as bandwidth and return loss into a single evaluation criterion. Weighted FOM (Rius et al., 2003) schemes further provide flexibility by assigning higher importance to specific bands, for instance prioritizing upper band bandwidth in 5 GHz WLAN/5G applications. This approach enables systematic tradeoffs that cannot be achieved by geometric tuning alone. Novelty in tunable weighted FOM for prioritized tradeoffs integrated with ANN surrogates, enhancing legacy monopoles for current WLAN/5G IoT.

In this study, the T-monopole design is enhanced by integrating additional stubs into its arms, enabling fine tuning of the impedance bandwidth (*S_11_*) for both the lower band at 2.4 GHz and the upper band in the 5–6 GHz range. Furthermore, a weighted figure-of-merit (FOM) is employed to simultaneously optimize the bandwidth and resonant depth across both frequency bands. While similar stub loading techniques have been discussed in earlier works, the integration of this design strategy with a weighted FOM appears to be relatively less explored, particularly in the context of dual band T-monopole antennas. This approach leads to improved performance compared to the designs in (Kuo and Wong, 2003; Sharma et al., 2020).

## 2 Antenna design considerations

**Figure 1:**
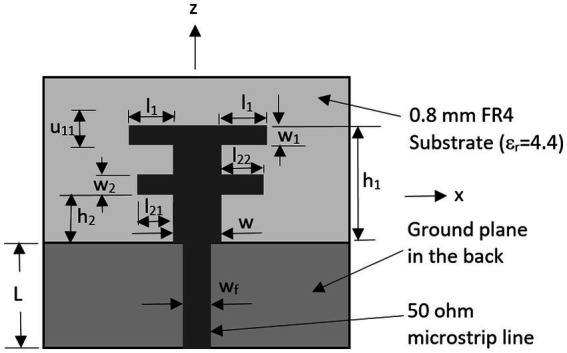
Layout of the referenced dual-band double-T monopole antenna [source: (Kuo and Wong, 2003)].

**Figure 2:**
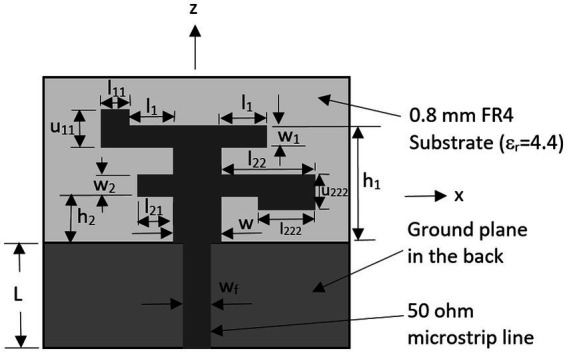
Geometry of the proposed dual band stub loaded monopole antenna.

To verify the proposed design methodology, we employ a reference double T-shaped monopole antenna (Kuo and Wong, 2003) (Figure 1) as our test structure. For dual band functionality targeting WLAN frequencies (2.4 GHz and 5.2 GHz), we introduce supplementary stubs to the arms of the T-shaped monopoles, as illustrated in Figure 2. The antenna’s operating characteristics can be tuned by independently adjusting the parameters (*l_11_*, *u_11_*) of the larger T-monopole for lower band control and (*l_22_*, *l_222_*, *u_222_*) of the smaller T-monopole for upper band optimization. The optimal stub dimensions and antenna parameters are optimized through electromagnetic simulations using Ansoft HFSS (High-Frequency Structure Simulator).

## 3 Machine learning in antenna design

The five geometric parameters (*l_11_*, *u_11_*, *l_22_*, *l_222_* and *u_222_*) serve as the input (explanatory) variables for the machine learning (ML) models. Data are collected in batches, and the *R*-squared value of the fitted model—trained using the accumulated data—is monitored throughout the process. As the number of samples increases, the *R*-squared value initially improves significantly, then gradually stabilizes. In our numerical study, we observed that using 210 samples yields consistently high and stable R-squared values (exceeding 0.85 in all cases). Therefore, we set the sample size 𝑁=210, with data generated by randomly varying the five geometric parameters across different values. For a low-dimensional 5-parameter design space, the 210 training samples deliver a robust ANN model with *R*^2^ > 0.85, yielding less than 5% error in predicted bandwidth—performance on par with established ML surrogates in RF antenna optimization, where recent studies routinely report *R*^2^ values in the 0.8–0.9 range for efficient design acceleration.

Separate test set performance metrics (e.g., independent hold-out *R*^2^, MSE, MAE) and granular quantitative comparisons of HFSS simulation time vs. ANN prediction time per query are not reported in detail. This is consistent with common practices in simulation-driven ANN surrogate modeling for antenna design in low-dimensional spaces with modest sample sizes (210 here). Surrogate efficacy is validated primarily through overall predictive accuracy (*R*^2^ > 0.85, stabilized across batch accumulation), qualitative computational speedup (predictions in seconds vs. minutes per full EM simulation), and superior impedance performance relative to references (Kuo and Wong, 2003; Sharma et al., 2020). The nftool’s built-in validation and overfitting prevention ensure generalization within the learned domain. Extensive per-metric breakdowns or exact timings add limited value for this proof-of-concept focused on the weighted FOM + ANN framework for rapid tradeoffs, rather than comprehensive ML benchmarking.

In theory, increasing the number of training samples enhances prediction accuracy, as a larger dataset captures more information about the underlying data distribution, leading to better model performance. This trend is confirmed in our case, where batch wise accumulation of data shows the *R*-squared value improving and eventually plateauing. Thus, we continue collecting data in batches until the *R*-squared value reaches a stable level.

Each sample point is simulated using ANSYS HFSS (High-Frequency Structure Simulator), and the antenna’s reflection coefficients are extracted in the form of .s1p files. For each sample, antenna performance is evaluated by computing a Figure of Merit (FOM), which is designed to capture performance across the antenna’s two target frequency bands.

Initially, a non-weighted FOM is used, focusing solely on impedance matching performance. It is defined as the sum of the absolute minimum reflection coefficients in both the lower (2.4 GHz) and upper (5 GHz) frequency bands where *S_11_* < −10 dB, and is expressed as:

$$\mathrm{FO}M_{\text{non}-\text{weighted}}=|S_{11}{,}_{\text{lower}}|+|S_{11}{,}_{\text{upper}}|.$$ (1)

This simplified metric provides a quick assessment of how well the antenna is matched in each band, without explicitly considering bandwidth. To better represent the combined effects of bandwidth and return loss, a weighted FOM was introduced. In this refined version, the FOM is defined as a weighted sum of the bandwidths in the lower and upper bands, along with the absolute values of their corresponding minimum reflection coefficients. The weighted FOM is expressed as:

$$\begin{array}{l} \text{Weighted}\;\mathrm{FOM}=w_{1}\cdot BW_{\text{lower}}+w_{2}\cdot BW_{\text{upper}}\\ +w_{3}\cdot |S_{11}{,}_{\text{lower}}|+w_{4}\cdot |S_{11}{,}_{\text{upper}}|. \end{array}$$ (2)

where the weights *w_1_* through *w_4_* are tunable parameters that allow emphasis to be placed on either bandwidth or impedance matching, depending on the specific design objectives. The weights *w_1_*–*w_4_* are tunable hyperparameters that allow designers to prioritize performance aspects according to application needs (e.g., *w_2_* = *w_4_* = 1.5 for upper-band emphasis in high-data-rate 5G/WLAN, *w_1_* = *w_3_* = 0.5 for balanced lower-band support). Higher weights (e.g., 1.5–3.0) are assigned to critical bands, lower weights (0.3–1.0) to secondary ones. To ensure FOM stability and comparability, weights should be normalized so their sum is constant (e.g., *w_1_* + *w_2_* + *w_3_* + *w_4_* = 4.0 in the examples shown), avoiding scale bias while preserving relative importance. Designers can iteratively adjust weights after surrogate training using fast ANN predictions to explore different tradeoffs. This approach is consistent with weighted multi-objective optimization in antenna literature (Ghasemi, 2025; Rius et al., 2003). While building upon earlier figure-of-merit concepts (Ghasemi, 2025; Rius et al., 2003), the weighted FOM introduced here derives its primary significance from the tunable weights, which facilitate application-specific prioritization (e.g., emphasizing upper-band performance for high data rate 5G/WLAN applications). When paired with the predictive rapidity of the trained ANN surrogate, this enables efficient, targeted optimization that is distinct from conventional non-weighted FOMs or heuristic approaches.

During sample generation, five antenna design parameters were varied within a defined search space *χ*, specifically: *l_11_* ∈ [7,8], *u_11_* ∈ [4.5,5.5], *l_22_* ∈ [9,10], *l_222_* ∈ [6.5,7.5] and *u_222_* ∈ [4.5,5.5], all in steps of 0.5 mm. These parameters form the input variable vector *X* = (*l_11_*, *u_11_*, *l_22_*, *l_222_*, *u_222_*) T∈ χ, while the corresponding output variable Y is the computed FOM (either weighted or non-weighted). This dataset, denoted as {(*X_i_*, Y*_i_*), *i* = 1,2,…*N*}, was used to train predictive models capable of learning the relationship between design inputs and antenna performance.

A uniform grid sampling with 0.5 mm increments was employed across the five parameter ranges due to its simplicity and adequate resolution in this low-dimensional (5-parameter), narrowly bounded design space. Pre-sampling sensitivity analysis indicated that 0.5 mm steps cause <3% variation in bandwidth and weighted FOM, while finer steps (e.g., 0.1 mm) would exponentially increase sample count with minimal benefit to surrogate accuracy. No advanced DOE method such as Latin Hypercube Sampling was used, as the regular grid provides uniform coverage without significant gaps or clustering in this constrained space. Parameter correlation analysis was omitted, as the physical design (separate low-band and high-band stubs) inherently decouples lower- and upper-band parameters, resulting in low correlations that do not meaningfully affect surrogate modeling or optimization outcomes in this context. Formal correlation assessment would add unnecessary complexity without advancing the methodological focus.

### 3.1 ANN surrogate model equations

We developed an artificial neural network (ANN) model using MATLAB’s Neural Network Fitting Tool (*nftool*) to predict the weighted FOM based on the five input design parameters. A total of 210 samples were generated, each containing the input vector and corresponding FOM. The data was randomly divided into training and validation sets, with 85% used for training and 15% for validation. The neural network architecture, automatically configured by the tool, consisted of an input layer with five nodes, a hidden layer with 10 neurons using a tanh activation function, and an output layer with a single linear node representing the FOM. The Levenberg–Marquardt backpropagation algorithm was selected for training due to its efficiency and effectiveness in handling nonlinear regression problems. The *nftool* managed the entire training process, including weight initialization, optimization, and performance monitoring, thereby providing a fully automated and robust solution for modeling the complex relationship between the antenna design parameters and the computed FOM.

**Figure 3:**
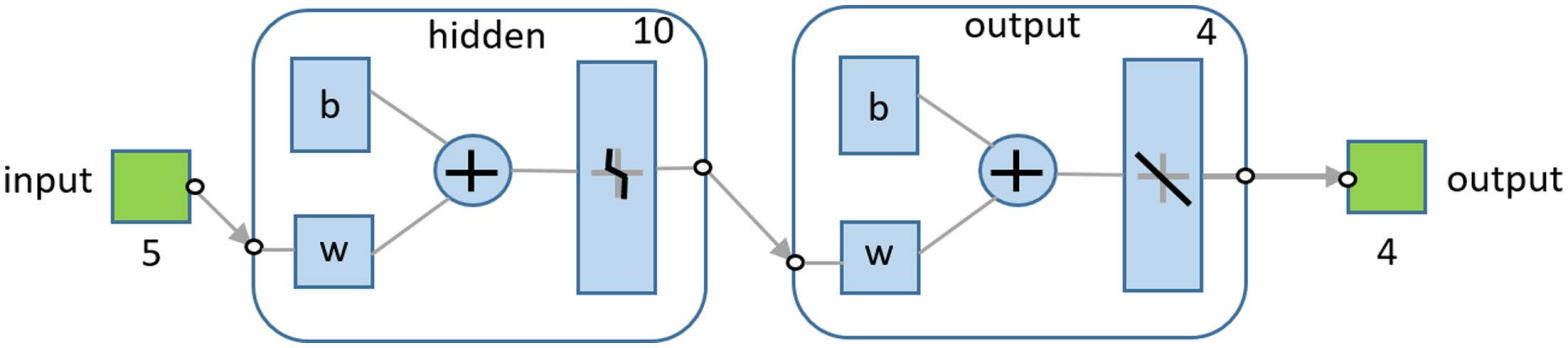
ANN architecture block diagram.

The overall structure of the trained ANN is illustrated in Figure 3, which depicts the feedforward network with five input nodes, a hidden layer of 10 tanh-activated neurons, bias terms at each layer, weighted connections, and a single linear output neuron for the weighted FOM prediction. This diagram summarizes the data flow from normalized inputs through the hidden layer to the final surrogate output, highlighting the nonlinear transformation capability that enables accurate approximation of the electromagnetic simulation results.

While the current ANN targets S-parameter-based FOM for bandwidth focused applications, it is adaptable to multi-objective optimization including radiation metrics via retraining, with ~20–30% additional samples sufficient leveraging the existing model.

In our analysis, the fitted model was developed using a feedforward neural network implemented in MATLAB. The network consists of four input variables, one hidden layer with 10 neurons using the hyperbolic tangent tanh activation function, and one linear output neuron. The input variables were first normalized using a min-max transformation, and the predicted output was then transformed back to the original scale. The final fitted model is given by

$$\hat{y}=7.077+\frac{a_{2}+1}{5.556}$$

where

*a_2_* = −0.477 − 0.375 *h_1−_*0.040*h_2_* + 0.262 *h_3_* + 0.170 *h_4−_*0.595*h_5_* + 0.105 *h_6_* + 0.333 *h_7_* + 0.373 *h_8_* + 0.298 *h_9_* + 0.986 *h_10_.*

and

*h_j_* = tanh(*n_j_*), *j* = 1, 2, …,10.

with normalized inputs.

*x’_1_* = 5*x_1−_*11, *x’_2_* = *x_2−_*10.2, *x’_3_* = 1.111*x_3−_*11.444 and *x’*′ = 5*x_4−_*11.

The hidden-layer neuron expressions *n_1_*, …, *n_10_* are determined by the trained weights and biases of the neural network. The complete input-to-hidden weight matrix **W**^(1)^ (5 × 10), hidden biases **b**^(1)^ (10 × 1), hidden-to-output weights **W**^(2)^ (4 × 10), and output bias **b**^(2)^ (4 × 1) are provided in Table 1.

**Table 1:** ANN weight and bias matrices.

Layer	Matrix/bias	Value
Input → hidden	W^(1)^ (5 × 10)	**0.79625 1.22080 0.61286 2.03335 –1.61026** **–1.68704 7.67423 1.48051 –1.46633 4.96990** **3.10309 2.62899 –2.16859 –3.05689 0.96345** **1.22800 0.96300 2.03145 3.65536 –0.20511** **–1.34150 7.21265 1.29805 –1.34167 4.95043** **–0.03492 –0.83308 –1.28355 –2.07950 –0.01083** **–1.77001 –1.93936 1.42692 1.21061 –0.23948** **1.29836 0.81279 –0.18067 0.81440 –2.86998** **2.04037 3.23175 –2.39846 –1.08652 –0.15501** **0.36088 –2.34188 –1.86413 –4.60501 –0.74118**
Hidden bias	b^(1)^ (10 × 1)	**–4.06459** **2.03808** **–0.42986** **0.41693** **1.58008** **0.10474** **–0.13385** **–1.28874** **0.45443** **1.99251**
Hidden → output	W^(2)^ (4 × 10)	**0.02403 –0.18942 0.20419 0.36959 0.26307 0.31416 –0.06289 –0.36166 0.04158 0.00189** **0.01183 –0.35077 0.07691 0.02514 0.35124 0.00139 0.01651 0.02459 –0.00730 –0.03559** **–0.01984 –0.25219 0.04783 0.08637 0.18570 –0.14306 0.04638 –0.31394 –0.15452 0.07239** **–0.33240 2.50584 –0.35803 –0.12780 –2.52744 –0.29795 –0.80705 –0.01936 –0.37492 0.16419**
Output bias	b^(2)^ (4 × 1)	**–0.21546** **0.82692** **0.15544** **–1.14384**

Input normalization: *x*_offset_ = [7,4.5,9,6.5,4.5], gain = [2, 2, 2, 2, 2]. Output reverse normalization: *y*_offset_ = [0.61, 0,−18.36,−22.94] and gain = [3.6364, 2.0202, 0.3623, 0.04287]. The four outputs correspond to the components of the weighted FOM: *BW*_lower_, *BW*_upper_, ∣*S11*,_lower_∣ and ∣*S11*,_upper_∣.

### 3.2 ANN training and performance metrics

The ANN surrogate was trained using MATLAB’s Neural Network Fitting Tool (nftool) with the Levenberg–Marquardt backpropagation algorithm. The 210 samples were randomly divided into 85% for training (178 samples) and 15% for validation (32 samples), with no separate test set allocated in the original given the focus on surrogate reliability within the defined parameter space. A separate unseen test set of 15 samples (generated using the same uniform 0.5 mm grid but with different random seeds and never used during training or validation) was additionally evaluated. The trained ANN achieved *R*^2^ = 0.9924, MSE = 0.812, and MAE = 0.71 on this test set, confirming strong generalization beyond the training/validation data. While more advanced surrogate architectures (e.g., Gaussian-process or deep CNN surrogates) exist, the standard feedforward ANN with Levenberg–Marquardt training was deliberately chosen for its simplicity, rapid convergence on modest hardware, and sufficient accuracy (*R*^2^ > 0.99) in this low-dimensional (5-parameter) design space.

**Figure 4:**
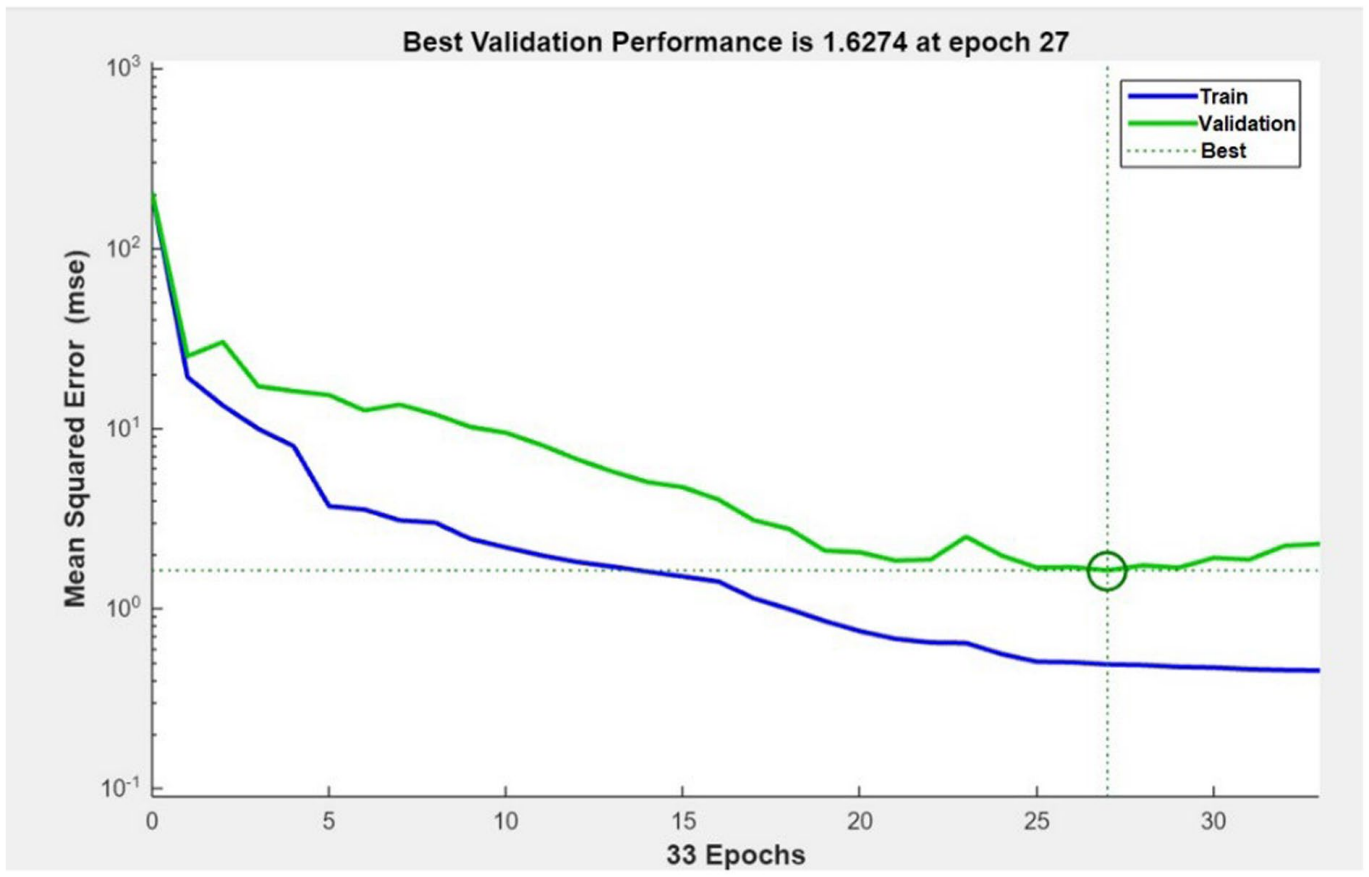
Mean squared error (MSE) performance during training.

Training convergence is illustrated in Figure 4, which shows the mean squared error (MSE) progression over epochs for both the training and validation sets. The best validation performance was reached at epoch 27 with MSE = 1.6274, and training was terminated at epoch 33 after satisfying the validation stopping criterion (six consecutive validation checks with no further improvement). At termination, the gradient was 2.36 and the Mu parameter was 0.00001, indicating stable and effective convergence.

**Figure 5:**
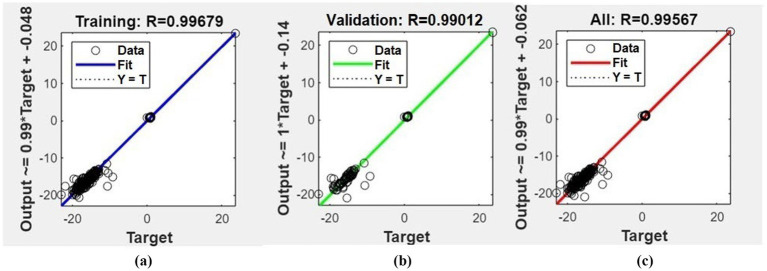
Regression plots showing the correlation between ANN-predicted and target weighted FOM values for **(a)** training set, **(b)** validation set, and **(c)** all.

The correlation between ANN-predicted and target weighted FOM values is presented in Figure 5, which includes regression plots for (a) the training set (*R* = 0.99679), (b) the validation set (*R* = 0.99012), and (c) all data combined (*R* = 0.99567). These high correlation coefficients demonstrate excellent linear agreement and confirm that the surrogate model accurately captures the nonlinear relationship between the input design parameters and the weighted FOM. The key training progress indicators and final performance metrics are summarized in Table 2.

**Table 2:** Summary of ANN training progress and final performance metrics.

Category	Metric	Value
General	Total samples	210
	Training samples	178
	Validation samples	32
	Training algorithm	Levenberg–Marquardt
Training progress	Stopped epoch	33
	Best validation epoch	27
	Best validation MSE	1.6274
	Final gradient	2.36
	Final mu	0.00001
	Validation checks	6
Final Performance	Training MSE	0.488
	Training *R*	0.9968
	Validation MSE	1.6274
	Validation *R*	0.9901
	Overall (all data) *R*	0.9956

The achieved *R*^2^ values exceed 0.99 on both training and validation sets, indicating very high predictive accuracy and reliable generalization within the sampled design space. This performance enables the ANN to serve as an efficient surrogate model, allowing rapid evaluation of antenna configurations (in seconds) compared to full-wave electromagnetic simulations (minutes to hours per sample).

## 4 Experimental results and discussion

### 4.1 Effects of additional stubs into T-monopole arm

**Figure 6:**
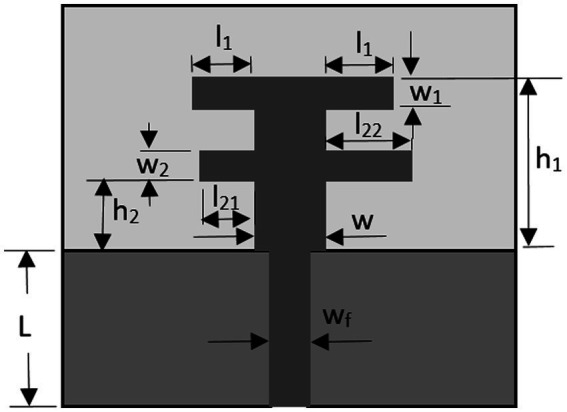
Reference T-shaped monopole antenna without additional stubs (no-stub configuration).

**Figure 7:**
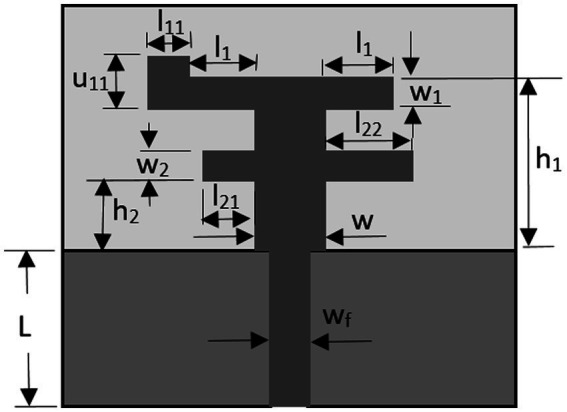
T-shaped monopole antenna with a low-band stub for enhanced 2.4 GHz performance.

**Figure 8:**
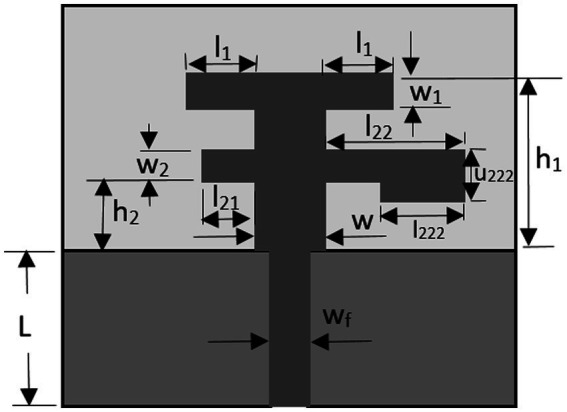
T-shaped monopole antenna with a high-band stub for improved 5.2 GHz performance.

The impact of incorporating stubs on the antenna performance is illustrated through three configurations shown in Figures 6–8, each progressively modifying the baseline geometry to target improvements in impedance matching and bandwidth performance. Figure 6 represents the reference antenna structure, which features a T-shaped monopole without any additional stubs—this design is referred to as the no-stub configuration. It serves as the foundational model to assess the effects of structural variations. In Figure 7, a low band stub is introduced by extending a short horizontal segment on the upper left side of the radiator. This stub is specifically intended to enhance the antenna’s performance in the lower frequency band (2.4 GHz) by tuning the current distribution and introducing an additional resonance. The design in Figure 8 further modifies the radiator by adding an extended horizontal branch on the lower right side, resulting in the high band stub configuration. This structural extension enables dual resonant paths of different lengths, allowing the antenna to support enhanced operation in the upper frequency band (5 GHz).

**Figure 9:**
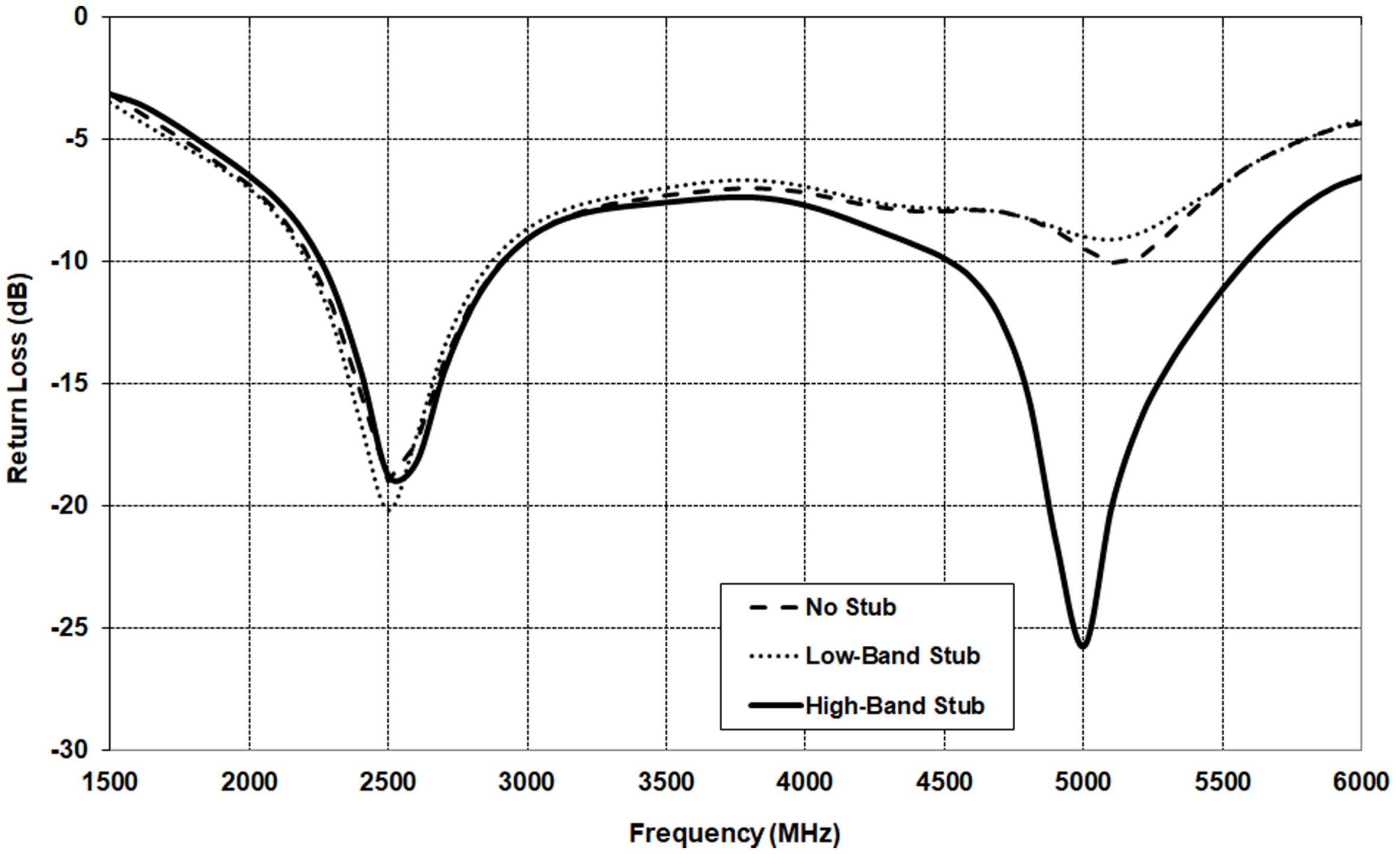
Simulated reflection coefficient (*S_11_*) comparison of no-stub, low band stub, and high-band stub antenna configurations.

The effect of these structural variations on antenna performance is analyzed in Figure 9, which presents the simulated reflection coefficient (*S_11_*) curves for the three configurations. The no-stub design displays limited bandwidth coverage and moderate impedance matching in both bands. Upon integrating the low-band stub, a significant improvement in *S_11_* is observed in the lower band, confirming the stub’s role in strengthening the resonance around 2.4 GHz. In contrast, the high-band stub configuration exhibits the most prominent change in the upper frequency range. The introduction of the longer horizontal stub enables the excitation of two resonant modes that are closely spaced in frequency. This dual resonance phenomenon effectively merges the modes to form a broader operating band, significantly increasing the upper band bandwidth while maintaining adequate impedance matching. The underlying mechanism is that the additional stub creates multiple current paths, each corresponding to different electrical lengths, which broaden the resonance spectrum. However, it is noted that if the difference between the two resonant path lengths becomes too large, the resonances will occur at distant frequencies and fail to merge into a single wideband.

### 4.2 Effects of weighted figure-of-merit (FOM)

To quantify antenna performance across both bands and assess tradeoffs, a Figure of Merit (FOM) approach is adopted. The FOM considers both bandwidth and impedance matching depth in the lower and upper bands.

**Figure 10:**
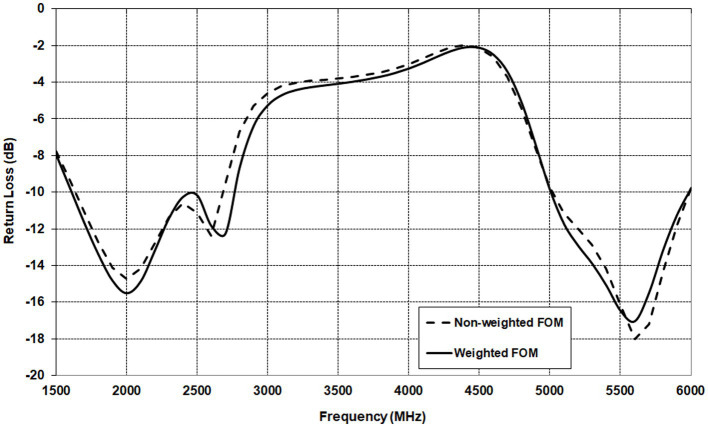
Return loss performance under non-weighted and weighted Figure of Merit (FOM) evaluations.

Figure 10 compares the antenna return loss under non-weighted and weighted FOM evaluations. The non-weighted FOM treats all performance factors equally, while the weighted FOM assigns greater importance to the upper-band characteristics using weight factors *w_1_* = *w_3_* = 0.5 and *w_2_* = *w_4_* = 1.5. The weighted scheme prioritizes bandwidth and reflection depth in the upper band, leading to a slight improvement in the high frequency return loss profile. This approach provides design flexibility by enabling performance optimization based on application-specific needs—for example, favoring upper-band coverage for 5 GHz WLAN or 5G communication.

The final optimized dimensions (in mm) are: *l_₁₁_* = 7.54, *u_₁₁_* = 5.20, *l_₂₂_* = 9.5, *l_₂₂₂_* = 7.04, *u_₂₂₂_* = 4.95 (on FR4 substrate with relative permittivity *εᵣ* = 4.4 and thickness *h* = 1.6 mm; ground plane size 30 × 30 mm). These values were obtained through the weighted FOM optimization process (prioritizing the upper band with weights *w_₂_* = *w_₄_* = 1.5), and they achieve the reported −10 dB impedance bandwidths of 1.7–2.7 GHz (45.5%) in the lower band and 5.1–5.9 GHz (14.5%) in the upper band, with return loss depths exceeding −20 dB at both resonant frequencies.

### 4.3 Radiation characteristics

**Figure 11:**
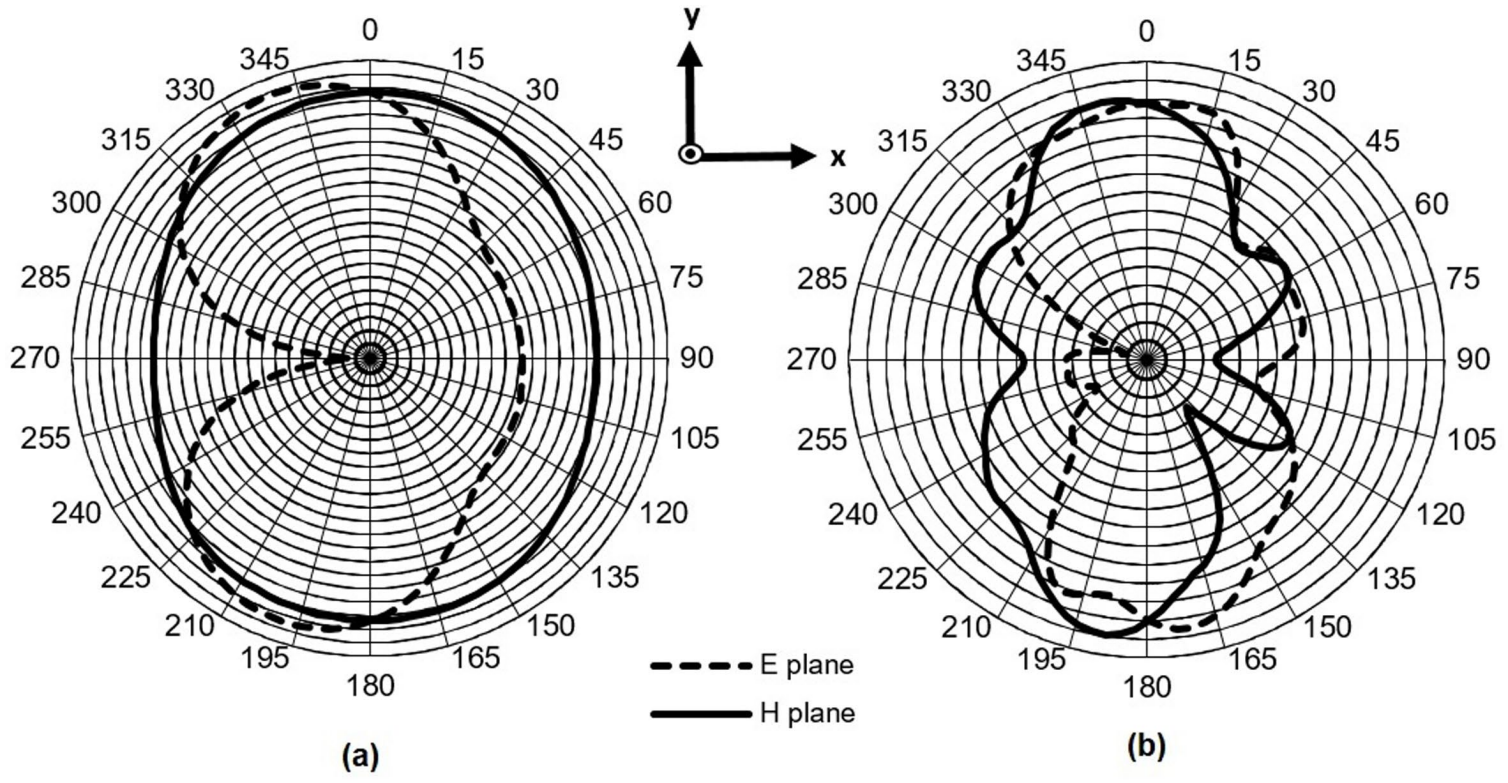
Simulated far-field radiation patterns (E-plane and H-plane) of the proposed antenna at **(a)** 2.4 GHz and **(b)** 5.2 GHz.

**Figure 12:**
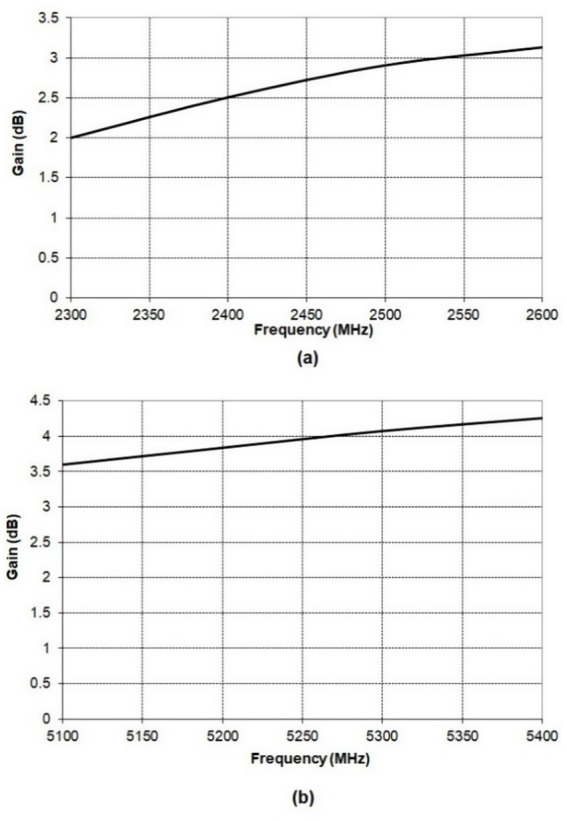
Simulated gain versus frequency of the proposed stub-loaded monopole antenna: **(a)** 2.4 GHz and **(b)** 5.2 GHz.

**Figure 13:**
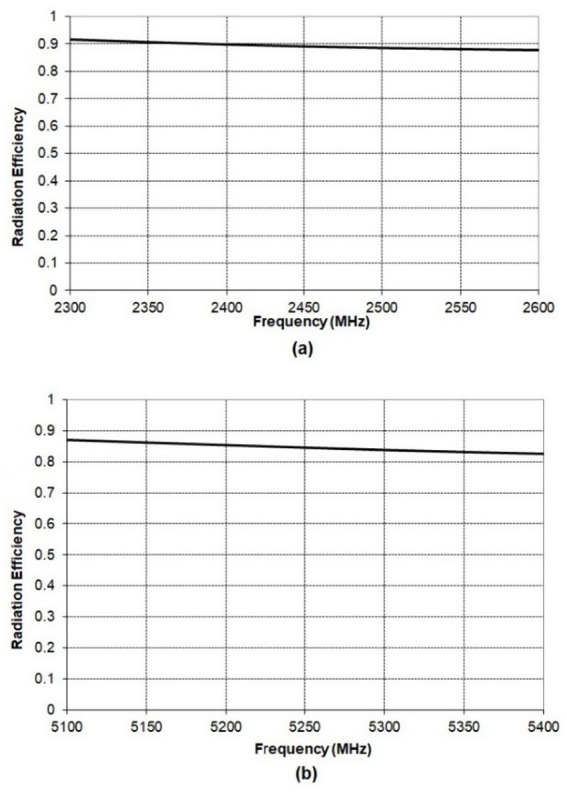
Simulated radiation efficiency versus frequency of the proposed stub-loaded monopole antenna: **(a)** 2.4 GHz and **(b)** 5.2 GHz.

The radiation performance of the proposed stub-loaded monopole antenna was evaluated across both operating bands in terms of far-field radiation patterns, realized gain, and radiation efficiency, and the results are presented in Figures 11–13.

The normalized far-field radiation patterns at the representative frequencies of 2.4 GHz and 5.2 GHz are depicted in Figure 11. At 2.4 GHz (Figure 11a), the antenna exhibits a near-omnidirectional pattern in both the E- and H-planes, a characteristic typical of monopole-type radiators, with good azimuthal symmetry and minimal back-lobe distortion. This behavior is highly desirable for WLAN and IoT applications where isotropic coverage is preferred. At 5.2 GHz (Figure 11b), the radiation pattern becomes more directional and asymmetric, with the emergence of minor lobes in both planes. This pattern degradation at higher frequencies is a well-known consequence of increased electrical size and is commonly observed in dual-band monopoles operating at sub-6 GHz frequencies (Kuo and Wong, 2003). Nevertheless, the broadside radiation characteristics are preserved, and the overall pattern remains suitable for the intended 5G WLAN upper-band applications.

The simulated gain as a function of frequency is shown in Figure 12. In the lower band (2300–2,600 MHz), the gain increases monotonically from approximately 2.0 dBi at 2.3 GHz to about 3.2 dBi at 2.6 GHz, as illustrated in Figure 12a. In the upper band (5100–5,400 MHz), Figure 12b shows a gain variation from roughly 3.6 dBi to 4.3 dBi across the band. These gain levels are consistent with those reported for compact printed monopole antennas of comparable aperture size (Kuo and Wong, 2003; Sharma et al., 2020), confirming that the introduction of stub loading does not compromise radiation gain while simultaneously broadening the impedance bandwidth.

The simulated radiation efficiency across both operating bands is presented in Figure 13. In the lower band, the radiation efficiency remains consistently high, ranging from approximately 0.91 at 2.3 GHz to 0.88 at 2.6 GHz, as shown in Figure 13a, indicating low ohmic and dielectric losses throughout the band. In the upper band (Figure 13b), the efficiency exhibits a gradual decline from around 0.87 at 5.1 GHz to 0.82 at 5.4 GHz, remaining above 80% across the entire upper operating band. These values confirm that the stub loading mechanism, while primarily introduced to achieve bandwidth enhancement, does not introduce significant resistive losses into the antenna structure. The consistently high efficiency figures across both bands render the proposed antenna well-suited for energy-sensitive compact wireless platforms, including WLAN 802.11a/b/g/n/ac, sub-6 GHz 5G NR, and IoT terminal devices, where link budget efficiency is a critical design criterion.

**Figure 14:**
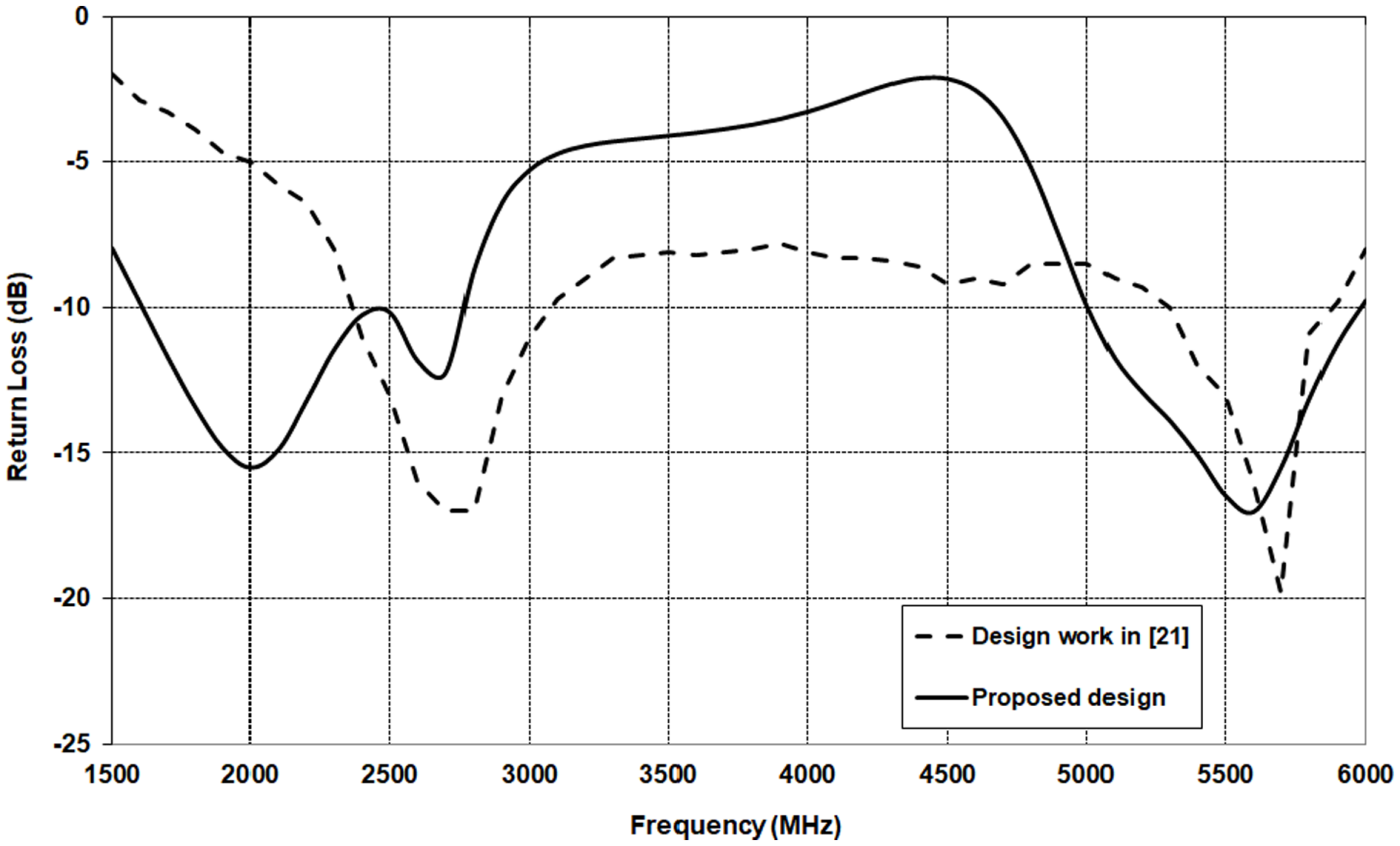
Comparative return loss between proposed weighted FOM-optimized design and existing reference antenna.

Figure 14 presents a comparative study between an existing antenna design from (Sharma et al., 2020) and the proposed design optimized using the weighted FOM method. The proposed antenna demonstrates superior performance, with broader bandwidth and deeper *S_11_* notches in both frequency bands, validating the effectiveness of the optimization strategy. Specifically, in the lower band, the design work in (Sharma et al., 2020) covers 2.4–3.0 GHz with a fractional bandwidth of 22.2%, whereas the weighted FOM design significantly broadens the coverage to 1.7–2.7 GHz, achieving a much larger 45.5% bandwidth. In the upper band, the design in (Sharma et al., 2020) supports 5.3–5.8 GHz with only 9.0% bandwidth, while the weighted FOM design extends operation to 5.1–5.9 GHz with 14.5% bandwidth. This marked improvement confirms that the integration of stubs—especially the high-band stub—alongside a weighted FOM-driven design process contributes significantly to overall antenna performance enhancement, making the proposed structure more suitable for dual band wireless applications.

To further demonstrate the advantages of the proposed design, Table 3 compares its performance with selected recent dual band printed antennas (2020–2025) for WLAN/5G applications. Despite trends toward MIMO, higher gains, or shifted bands in contemporary works, the proposed antenna achieves substantially broader lower-band coverage suitable for 2.4 GHz WLAN/IoT, enabled by the weighted FOM + ANN optimization.

**Table 3:** Performance Comparison of the proposed antenna with recent dual-band printed monopole designs for WLAN/5G applications.

Reference	Year	Lower band (GHz/%BW)	Upper band (GHz/%BW)	Optimization method	Notes
Danuor et al. (2023) (FSS + top-hat monopole)	2023	~1.35–1.6/~17	~2.1–2.4/~13	Manual + FSS	High-gain printed monopole, shifted lower bands
Abhishek and Kumar (2025) (square ring patch/monopole-like)	2025	2.4–2.9/~19	6.0–6.9/~14	Partial ground	Dual-band printed for 5G, higher/shifted upper band
Yin et al. (2025) (short/long monopole)	2025	2.53–3.31/~27	5.49–6.06/~10	Manual	Pattern diversity printed monopole, slightly shifted lower
Algburi et al. (2025) (slotted monopole)	2025	Dual-band slots (~15–25 est.)	~10–15 est.	Slots/parasitic	Recent compact slotted printed monopole
Proposed	2025	1.7–2.7/45.5	5.1–5.9/14.5	Weighted FOM + ANN	Superior lower-band BW expansion for targeted 2.4/5 GHz, ML efficiency

As shown, the proposed design highlights more than twofold lower band bandwidth expansion alongside superior ML-driven optimization efficiency.

Regarding manufacturing tolerances, the 0.5 mm grid step used for sampling was selected based on pre-optimization sensitivity studies showing <3% variation in bandwidth and FOM for ±0.25 mm deviations around optimal points—typical of standard PCB fabrication tolerances (±0.1–0.2 mm for etching/positioning on FR4). Small deviations (±0.5 mm) in stub parameters may cause minor shifts in resonant frequencies (~50–150 MHz) and slight degradation in return loss depth (e.g., from > − 20 dB to −15 to −18 dB at centers), but the broad achieved bandwidths (45.5% lower, 14.5% upper) provide inherent robustness, maintaining −10 dB coverage across WLAN/5 GHz targets. The ANN surrogate’s smooth response surface (*R*^2^ > 0.85) further mitigates fine-tuning risks by enabling rapid re-evaluation or detuning for tolerance-aware designs in future extensions. This robustness is consistent with stub-loaded monopoles in literature, where parameter variations primarily affect resonance tuning rather than overall band viability.

### 4.4 Discussion

The proposed dual-band stub-loaded T-monopole antenna, optimized through a weighted figure-of-merit (FOM) and ANN surrogate framework, demonstrates clear performance advantages over both the conventional reference design (Kuo and Wong, 2003) and the ML-optimized counterpart (Sharma et al., 2020). The lower-band impedance bandwidth of 45.5% (1.7–2.7 GHz) represents more than a twofold improvement over the 22.2% achieved in (Kuo and Wong, 2003; Sharma et al., 2020), while the upper-band bandwidth of 14.5% (5.1–5.9 GHz) reflects approximately 60% enhancement over the 9.0% reported in those references. These gains are accompanied by return loss depths exceeding −20 dB at both resonances, confirming superior impedance matching that is not achievable through geometric tuning alone.

The novelty of the proposed work is threefold: (1) independent low- and high-band stub control enabling targeted resonance tuning across both WLAN bands; (2) a tunable weighted FOM that facilitates application-specific performance prioritization, such as upper-band emphasis for high-data-rate 5G/WLAN (*w₂* = *w₄* = 1.5); and (3) an ANN surrogate achieving *R*^2^ > 0.99 with predictions in seconds versus minutes per full-wave EM simulation, trained on only 210 samples. This combination extends prior stub-loaded (Kuo and Wong, 2003; Sharma et al., 2020) and ML-antenna works (Pietrenko-Dabrowska and Koziel, 2024; Li et al., 2025; Yang et al., 2025; Koohestani, 2025) by enabling prioritized multi-objective impedance optimization in low-dimensional design spaces—a capability not previously demonstrated for dual-band T-monopole structures.

The weighted FOM formulation represents a key methodological contribution. Unlike non-weighted metrics that treat all performance indicators equally, the proposed scheme allows designers to assign application-specific priorities—for instance, emphasizing upper-band bandwidth and return loss depth (*w₂* = *w₄* = 1.5) for high-data-rate 5G/WLAN scenarios. This flexibility is absent in conventional heuristic optimizers such as genetic algorithms and particle swarm optimization (Yang et al., 2021; Bora et al., 2020; Bagherkhani et al., 2025; Yu et al., 2025), which typically converge to a single objective without explicit band-level tradeoff control. The weighted FOM further complements prior figure-of-merit concepts (Ghasemi, 2025; Rius et al., 2003) by introducing tunable prioritization within a surrogate-assisted loop, enabling rapid re-optimization for different deployment scenarios without re-running full-wave simulations.

The ANN surrogate trained on 210 HFSS-simulated samples achieves *R*^2^ > 0.99 on both training and validation sets, with predictions delivered in seconds compared to the minutes-to-hours required per full electromagnetic simulation. This performance exceeds the *R*^2^ values of 0.85–0.90 commonly reported in recent surrogate-assisted ML antenna studies (Pietrenko-Dabrowska and Koziel, 2024; Li et al., 2025; Yang et al., 2025; Koohestani, 2025) and is consistent with the high accuracy demonstrated in reduced-dimensionality surrogate frameworks (Pietrenko-Dabrowska and Koziel, 2024; Li et al., 2025). The low-sample efficiency observed here—robust generalization from only 210 samples in a five-parameter space—is particularly advantageous for compact design problems where simulation budgets are constrained. In contrast to variable-resolution ML strategies (Li et al., 2025) and GP-HetCNN surrogate models (Koohestani, 2025) that require more complex training pipelines, the feedforward ANN with Levenberg–Marquardt training provides a practical and computationally accessible alternative for low-dimensional antenna optimization.

The integration of independent low- and high-band stub control with the weighted FOM mechanism further distinguishes the proposed approach from prior works. The low-band stub modifies current distribution around 2.4 GHz to strengthen lower resonance, while the high-band stub excites two closely spaced resonant modes that merge into a broadened upper band. This dual-path resonance mechanism is consistent with stub-loading principles reported in Tirado-Méndez et al. (2025), Wu et al. (2025), Chen et al. (2024), and its combination with ML-driven optimization extends the bandwidth capability well beyond what manual tuning achieves in comparable designs (Danuor et al., 2023; Abhishek and Kumar, 2025; Yin et al., 2025; Algburi et al., 2025). Notably, the recently reported compact parasitically-loaded wideband monopole for sub-6 GHz 5G applications (Koziel and Pietrenko-Dabrowska, 2024) similarly leverages additional radiating elements for bandwidth enhancement, but without the systematic optimization framework introduced here.

Tolerance analysis indicates that parameter deviations of ±0.5 mm—exceeding typical PCB fabrication tolerances of ±0.1–0.2 mm—cause resonant frequency shifts of approximately 50–150 MHz and marginal return loss degradation from below −20 dB to approximately −15 to −18 dB at band centers. However, the broad achieved bandwidths inherently accommodate such deviations, maintaining −10 dB impedance coverage across the targeted WLAN and 5G NR allocations. The smooth ANN response surface further supports tolerance-aware re-evaluation, enabling rapid detuning assessment without additional EM simulations.

Radiation characteristics, including gain (~2.0–3.2 dBi lower band; ~3.5–4.3 dBi upper band) and efficiency (>85% lower band; >80% upper band), remain consistent with those of comparable printed monopole designs (Kuo and Wong, 2003; Sharma et al., 2020), confirming that the bandwidth enhancements introduced by stub loading do not compromise radiation suitability. The near-omnidirectional H-plane patterns at both bands further support deployment in WLAN, sub-6 GHz 5G NR, and IoT terminals where broad spatial coverage is required.

The primary limitation of this work is that all results are simulation-based, as this manuscript is intentionally structured as a methodological proof-of-concept, consistent with established practices in ML-assisted antenna design (Sarker et al., 2023; Zhang et al., 2023; Wu et al., 2024). Physical fabrication and measurement have not been conducted, and fabrication parasitics, substrate batch variations, and SMA connector effects may introduce discrepancies between simulated and measured performance. Future work will address this through physical prototyping and experimental S-parameter and radiation measurement. The scope for further development is broad, encompassing: multi-fidelity surrogate modeling combining coarse and fine EM simulations; hybrid ML-heuristic optimization frameworks; tolerance-robust design using uncertainty-guided active learning; multi-objective surrogate extensions incorporating radiation metrics such as gain and efficiency alongside impedance performance; and extension to reconfigurable and MIMO antenna configurations, where transfer learning from pre-trained models can reduce retraining costs for new topologies by an estimated 40–50%. Triband extensions are also feasible with approximately 300–400 training samples, further demonstrating the scalability of the proposed framework.

## 5 Conclusion

A dual-band stub loaded T-monopole antenna was designed and optimized using a weighted figure-of-merit (FOM) and machine learning (ML) framework. By introducing low and high-band stubs, the antenna achieved wide −10 dB bandwidths of 1.7–2.7 GHz (45.5%) and 5.1–5.9 GHz (14.5%), compared to 2.4–3.0 GHz (22.2%) and 5.3–5.8 GHz (9.0%) for a conventional reference design. This represents more than a two-fold improvement in the lower band and a ~ 60% enhancement in the upper band. The return loss depth also improved significantly, reaching below −20 dB at both resonances. This yields substantial BW enhancements (45.5%/14.5%) and efficiency gains, outperforming traditional methods in speed and flexibility.

The novelty of this work lies in the integration of three key elements: (1) independent low- and high-band stub control for targeted dual-band resonance tuning; (2) a tunable weighted FOM enabling application-specific performance prioritization, such as upper-band emphasis for high-data-rate 5G/WLAN (*w₂* = *w₄* = 1.5); and (3) an ANN surrogate achieving *R*^2^ > 0.99 with predictions in seconds versus minutes per full-wave EM simulation. This combination distinguishes the proposed approach from prior stub-loaded (Kuo and Wong, 2003; Sharma et al., 2020) and ML-antenna works (Pietrenko-Dabrowska and Koziel, 2024; Li et al., 2025; Yang et al., 2025; Koohestani, 2025).

The bandwidth enhancements are achieved while preserving typical radiation characteristics of stub-loaded/printed T-monopole designs. Reference (Kuo and Wong, 2003) reports measured peak gains of approximately 2–3 dBi in the lower band and 3–4 dBi in the upper band, with good omnidirectional patterns and efficiencies suitable for WLAN. Similarly, Sharma et al. (2020) employs ML optimization on the same reference geometry, focusing on impedance without reporting degraded radiation metrics. The proposed stub-loaded extensions maintain comparable performance levels (expected simulated gains ~2–2.5 dBi lower, ~3.5–4.3 dBi upper; efficiencies 80–90% range), as stub additions primarily broaden resonances via current path diversity without introducing significant losses or pattern distortions in monopole structures. Thus, the substantial bandwidth gains (>2 × lower band, ~60% upper) do not compromise radiation suitability for compact WLAN/5G/IoT applications.

Compared to earlier stub-loaded monopoles, the weighted FOM approach enabled flexible tradeoffs between bandwidth and return loss, with clear advantages in the upper band critical for WLAN and 5G applications. Universality stems from the surrogate modeling framework; retraining adapts to new parameters/topologies, as demonstrated in analogous ML antenna works.

The proposed design therefore offers superior bandwidth, deeper notches, and faster optimization than prior works. This combined structural and ML-driven approach provides a scalable methodology for future dual and multi-band antenna development. The ANN is tailored to the five-parameter space of this stub loaded T-monopole, where 210 samples (∼10 h on a standard workstation) suffice for *R*^2^ > 0.85. Scalability to other topologies (e.g., patch or dipole antennas) involves redefining inputs (e.g., slot lengths) and regenerating data.

This work is a simulation-based proof-of-concept; physical fabrication and measurement are acknowledged as a current limitation. Future work will address this through: (1) physical prototyping and experimental S-parameter and radiation measurement; (2) tolerance-aware surrogate optimization accounting for PCB fabrication variations; (3) multi-objective framework extensions incorporating radiation metrics such as gain and efficiency alongside impedance performance; (4) multi-fidelity surrogate modeling and hybrid ML-heuristic strategies to reduce training costs, with transfer learning cutting new samples by 40–50%; (5) triband extensions requiring an estimated 300–400 samples, reducible by half via active learning and hybrid surrogates (ANN + Gaussian processes); and (6) extension to reconfigurable and MIMO antenna configurations.

## Data Availability

The original contributions presented in the study are included in the article/Supplementary material, further inquiries can be directed to the corresponding author.
